# Thrombin Generation as a Method to Identify the Risk of Bleeding in High Clinical-Risk Patients Using Dual Antiplatelet Therapy

**DOI:** 10.3389/fcvm.2021.679934

**Published:** 2021-06-10

**Authors:** C. P. D. M. de Breet, S. Zwaveling, M. J. A. Vries, R. G. van Oerle, Y. M. C. Henskens, A. W. J. van't Hof, P. E. J. van der Meijden, L. Veenstra, H. ten Cate, R. H. Olie

**Affiliations:** ^1^Department of Internal Medicine, Maastricht Universitair Medisch Centrum+, Maastricht, Netherlands; ^2^Department of Internal Medicine, Zuyderland Medisch Centrum, Heerlen, Netherlands; ^3^Department of Biochemistry–CARIM, Maastricht University, Maastricht, Netherlands; ^4^Groene Hart Ziekenhuis, Gouda, Netherlands; ^5^Department of Internal Medicine, Jeroen Bosch Ziekenhuis, ‘s-Hertogenbosch, Netherlands; ^6^Department of Cardiology, Zuyderland Medisch Centrum, Heerlen, Netherlands; ^7^Department of Cardiology, Maastricht Universitair Medisch Centrum+, Maastricht, Netherlands

**Keywords:** thrombin generation, dual antiplatelet therapy, bleeding risk, percutaneous coronary intervention, coagulation factors

## Abstract

**Background:** Patients using dual antiplatelet therapy after percutaneous coronary intervention are at risk for bleeding. It is currently unknown whether thrombin generation can be used to identify patients receiving dual antiplatelet therapy with increased bleeding risk.

**Objectives:** To investigate whether thrombin generation measurement in plasma provides additional insight into the assessment of bleeding risk for high clinical-risk patients using dual antiplatelet therapy.

**Methods:** Coagulation factors and thrombin generation in platelet-poor plasma were measured in 93 high clinical-risk frail patients using dual antiplatelet therapy after percutaneous coronary intervention. During 12-month follow-up, clinically relevant bleedings were reported. Thrombin generation at 1 and 6 months after percutaneous coronary intervention was compared between patients with and without bleeding events.

**Results:** One month after percutaneous coronary intervention, the parameters of thrombin generation, endogenous thrombin potential, peak height, and velocity index were significantly lower in patients with bleeding in the following months compared to patients without bleeding. At 6 months follow-up, endogenous thrombin potential, peak height, and velocity index were still (significantly) decreased in the bleeding group as compared to non-bleeders. Thrombin generation in the patients' plasma was strongly dependent on factor II, V, and VIII activity and fibrinogen.

**Conclusion:** High clinical-risk patients using dual antiplatelet therapy with clinically relevant bleeding during follow-up show reduced and delayed thrombin generation in platelet-poor plasma, possibly due to variation in coagulation factors. Thus, impaired thrombin-generating potential may be a “second hit” on top of dual antiplatelet therapy, increasing the bleeding risk in high clinical-risk patients. Thrombin generation has the potential to improve the identification of patients using dual antiplatelet therapy at increased risk of bleeding.

## Introduction

Each year in Europe, about 3.6 million patients receive dual antiplatelet therapy (DAPT) during 6 to 12 months after a percutaneous coronary intervention (PCI) (1). This patient population has an increased risk for both bleeding and ischemic events. Risk factors for bleeding and ischemic events are multifactorial, and it remains difficult to predict the individual bleeding vs. ischemic risk. Known risk factors for the occurrence of bleeding complications are, among others, low on-treatment platelet reactivity, the use of chronic oral anticoagulation therapy and older age (2, 3). On the other hand, ischemic events after PCI are associated with initial presentation with acute coronary syndrome (ACS), the stent itself (in-stent thrombosis), older age, diabetes, and chronic kidney disease (4). Identification of patients who are at higher risk of bleeding or ischemic events could lead to better personalized treatment and reduce the burden of adverse events.

While DAPT substantially reduces the risk of ischemic events, it comes with an impairment of primary hemostasis (5, 6). However, the overall hemostatic potential is determined not only by platelet reactivity but also by other factors like endothelial cell barrier integrity and the coagulation system including multiple coagulation factors (7). Variation in coagulant activity therefore impacts the bleeding risk in patients with a platelet function impairment inflicted by DAPT. This “second hit” of coinciding coagulation impairment can be due to variation in specific plasma components, like the level of factor VIII that shows substantial variation in the population (8), or DAPT-dependent reduction in the availability of the platelet procoagulant surface for efficient assembly of coagulation factor complexes promoting the formation of thrombin (9–11). Both mechanisms can be addressed in thrombin generation (TG) measurements using the “calibrated automated thrombogram” (CAT) (12). Thus far, a number of studies have demonstrated TG as a valid tool to detect a bleeding tendency in patients with hemophilia A and B, von Willebrand disease, factor XI deficiency, platelet disorders, and vitamin K antagonist (VKA) treatment (13–17).

One would expect that a reduction of platelet activation and aggregation by antiplatelet therapy leads to a diminished TG, measured in platelet-rich plasma (PRP) or whole blood (WB). Indeed, studies confirmed that treatment with aspirin, clopidogrel, or both reduced TG parameters in PRP and WB (18–23). The relationship between TG and the risk of bleeding in case of DAPT is rather underexposed. Therefore, in the present study, we investigated the potential use of the TG assay for the assessment of bleeding risk in high clinical-risk patients using DAPT. Besides, we were interested whether bleeding risk is influenced by the level of coagulation factors (24). TG in this study was measured in PPP, since this is by far the most used and standardized method of TG measurement. Hence, we hypothesized that patients who suffer from clinically relevant bleedings during DAPT show lower TG parameters compared to patients without bleeding events.

## Materials and Methods

### Study Population and Design

The current study is a sub-study of a prospective cohort study in the Maastricht University Medical Center+ (MUMC+), monitoring high clinical-risk patients on dual/triple antithrombotic therapy after PCI, as previously described (25). DAPT includes a combination of low-dose aspirin with either clopidogrel, ticagrelor, or prasugrel. DAPT duration was either 6 or 12 months, dependent on the PCI indication: elective vs. acute (ACS), respectively. In this exploratory study, patients were included *via* the outpatient clinic of the Thrombosis Expertise Center in the MUMC+, during a period of 2 years.

All PCI patients were screened by one dedicated cardiologist, and, after informed consent was obtained, high clinical-risk patients were included within 1 month after PCI. High clinical-risk patients in the prospective cohort study were defined as patients who had ≥3 risk factors for bleeding or ischemic events, which include female gender, hypertension, anemia at time of PCI (<13.2 g/dl for men, <11.8 g/dl for women), age >75 years, previous stroke, previous major bleeding, renal dysfunction [estimated glomerular filtration ratio (eGFR) <60 ml/min], known hepatitis or liver transplant, triple antithrombotic therapy, previous gastric ulcers, diabetes mellitus, low body weight (<60 kg), use of non-steroidal anti-inflammatory drugs or selective serotonin reuptake inhibitors, previous in-stent thrombosis, and/or high-risk stenting (multivessel PCI or main coronary artery stenting). The cohort population included patients who met the criteria named above, had been fasting for 4 h before blood withdrawal, and had a PCI in the past 1–2 months. Patients were excluded in case of non-compliance. The latter was checked during the visit by interview and by contacting the pharmacy to confirm dispensing of the drugs. According to the study protocol, TG was measured in the first group of 200 patients included in the prospective cohort study. Specific inclusion criteria for this sub-study were usage of a combination of low-dose aspirin (80–100 mg) and a P2Y12 inhibitor (clopidogrel 75 mg, prasugrel 5–10 mg, or ticagrelor 90 mg) (DAPT) for a planned duration of >6 months, and patients for whom TG measurements were available. An additional exclusion criterion for this sub-study was the use of vitamin K agonists, direct oral anticoagulants, or low-molecular-weight heparins. All patients provided written consent. Ethical approval was obtained from the medical ethical committee of the MUMC+ [(METC aZM/UM), approval number: 11-2-096].

### Follow-Up and Study Endpoints

**Figure 1:**
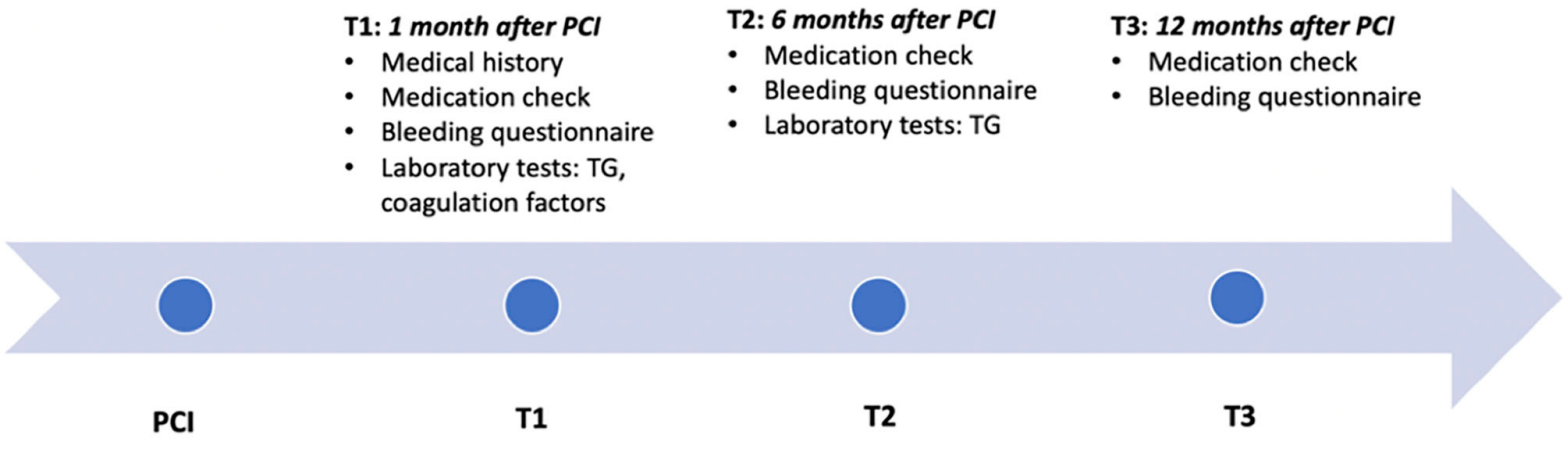
Timeline. Timeline of the study, showing the three contact moments: TI, T2, and T3, at respectively, 1, 6, and 12 months after PCI. The collected information and performed tests at each consultation is described. TG, Thrombin generation; PCI, percutaneous coronary intervention.

After referral, consultation at the thrombosis expertise center and study visits were planned at 1 month (T1) and 6 months (T2) after PCI (Figure 1). Twelve months after PCI (T3) patients using DAPT >6 months were contacted by phone for final follow-up. Blood was drawn at T1 and once again at T2 in case DAPT was continued for a total treatment duration of 9–12 months. During all three contact moments, a thorough history on minor and major bleeding was taken using the International Society on Thrombosis and Haemostasis (ISTH) Bleeding Assessment Tool (ISTH-AT). Using these structured questionnaires, bleeding events were recorded and categorized according to the definition of the Bleeding Academic Research Consortium (BARC), which contains unified and validated bleeding criteria (26–28). The primary endpoint of this study was clinically relevant bleeding during DAPT treatment, defined as BARC type 2, 3, or 5. Bleeding events presented at T1 include bleedings from PCI until T1, at T2 from T1 until T2, and at T3 from T2 until T3 (Figure 1). To investigate whether TG could identify patients with future clinically relevant bleedings, all bleeding events recorded at T2 and T3 were included in the analysis for TG measured at T1, and bleeding events recorded at T3 for TG measured at T2. In addition, during the follow-up visits, medical history, recurrent ischemic events, concomitant medication, intoxications, and compliance were recorded. To minimize recall bias, this information was collected in conversation with the patient and cross-checked with the hospital's electronic medical records.

### Blood Samples

During the first and second study visit, venous blood was collected through separate antecubital venipuncture in Vacuette® tubes (Greiner Bio-One, GmbH, Kremsmünster, Austria) containing sodium citrate (3.2%). PPP was prepared by two centrifugation steps at 2,000 *g* for 10 min and aliquots were stored at −80°C. TG was measured in all samples, simultaneously.

### Reagents

Innovin (Dade-Behring, Marburg, Germany) was used as a source of recombinant tissue factor (TF). Synthetic phospholipids (PL), consisting of phosphatidylserine, phosphatidylethanolamine, and phosphatidylcholine (1:1:3, mol:mol:mol), were from Avanti Polar Lipids Inc. (Alabaster, AL, USA). Z-Gly-Gly-Arg-aminomethylcoumarine (ZGGR-AMC) was purchased from Bachem (Basel, Switzerland). The calibrator, α2-macroglobulin-thrombin complex, was prepared as described by Hemker et al. (12). Hepes buffers containing 5 mg/ml or 60 mg/ml bovine serum albumin were prepared as described previously (29).

### Thrombin Generation

TG in PPP was measured by calibrated automated thrombogram (CAT) in triplicate, as previously described (30). In short, the reaction was initiated with 20 μl of stimulus [tissue factor (TF)], containing a final concentration of 1 pM TF and 4 μM phospholipids (PL). Next, 80 μl of plasma was added to the well. The calibration wells contained 20 μl of calibrator, with a final thrombin concentration of 100 nM. The reaction was started by adding 20 μl of FluCa (416.7 μM ZGGR-AMC and 16.7 mM CaCl_2_ in BSA60 buffer). To minimize variation, samples of normal pool plasma were measured on each plate. Data were analyzed using software from Thrombinoscope version 5.0 (Maastricht, the Netherlands). Low TF concentration was used to include the contribution of the intrinsic pathway factors VIII, IX, and XI (31, 32). The analyzed TG parameters for all samples were lag time, Endogenous Thrombin Potential (ETP), velocity index, peak level of thrombin formation, and time to peak.

### Additional Analyses

Measurements of coagulation factors were performed using the STA-R Evolution analyzer (Stago, Asnières sur Seine, France). Factor (F)II, V, VII, VIII, IX, X, XI, and XII activity levels were assessed with clotting assays triggered by either a thromboplastin-based reagent (FII, FV, FVII, and FX) or a kaolin-based reagent (FVIII, FXI, FIX, and FXII). Fibrinogen levels were measured using the Clauss method, and both fibrinogen and von Willebrand factor (vWF) were measured with the CS2100i analyzer from Sysmex (33).

### Statistical Analysis

Statistical analysis was performed using SPSS software (version 24). Patients with missing data (other than TG) were not excluded from the analysis. Normal distribution of all continuous variables was calculated using the Kolmogorov–Smirnov and Shapiro–Wilk Test of Normality combined with evaluation of the histograms. For normally distributed continuous variables, differences between groups were tested by Student's *t*-test and represented by means and standard deviations (SD). For non-normally distributed continuous variables, differences between groups were tested by Mann–Whitney *U* test and represented by medians and interquartile range (IQR) or range (when subgroups where too small for an IQR). Categorical variables were tested by Chi-square test and represented as numbers and percentages.

A multiple regression analysis was done to investigate coagulation factors as determinants of thrombin generation. For each model, the adjusted *R*^2^ and the standardized regression coefficients (beta) of the independent variables (coagulation factors) were calculated. A two-sided *p* ≤ 0.05 was considered statistically significant.

## Results

### Study Population

**Figure 2:**
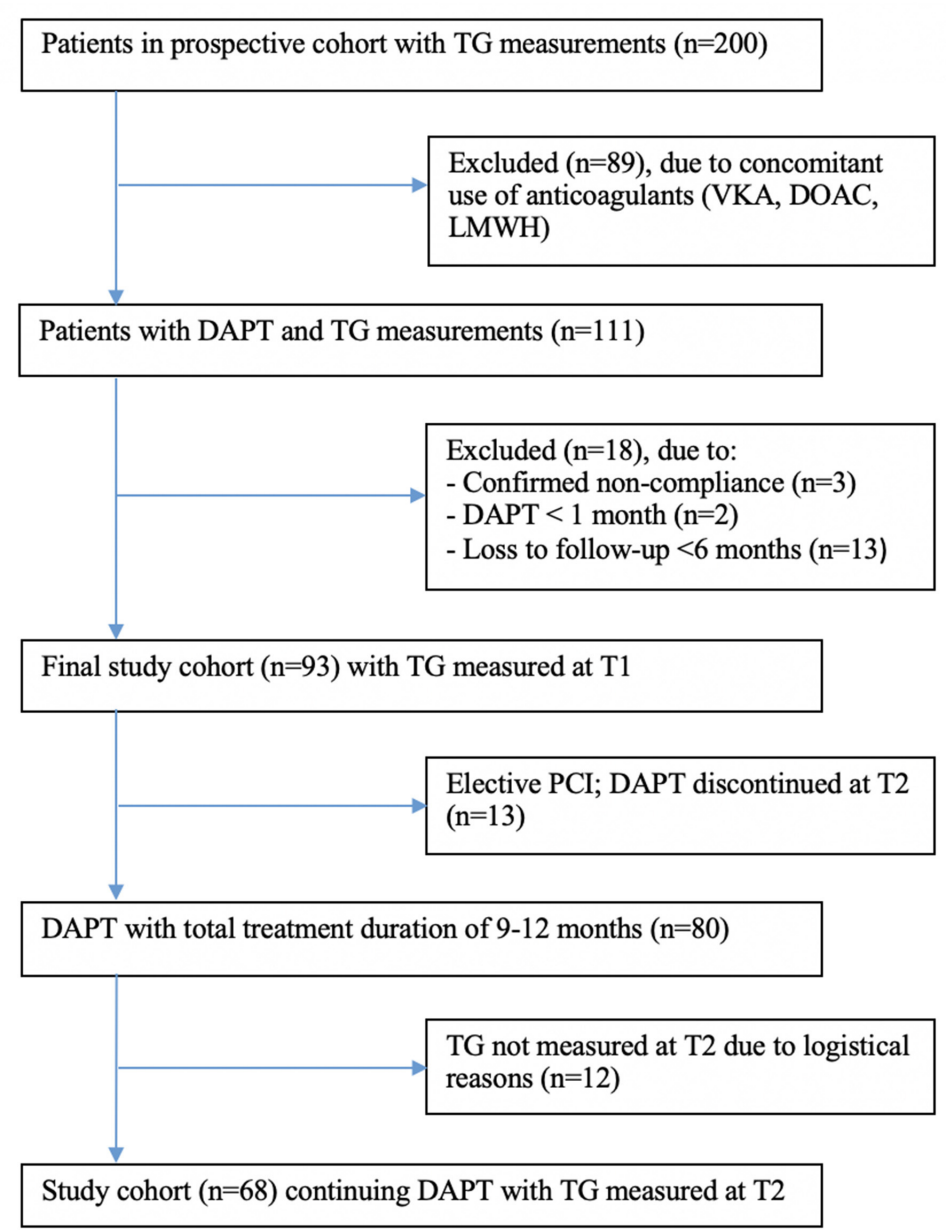
Flow diagram of study inclusion and follow-up. TG, thrombin generation; VKA, vitamin K antagonist; DOAC, direct-acting oral anticoagulants; LMWH, low molecular weight heparin; DAFT, dual antiplatelet therapy; PCI, percutaneous coronary intervention.

In total, 111 patients were eligible for this sub-study, of whom 18 patients had to be excluded for several reasons: 3 patients were not compliant, 2 patients used DAPT <1 month, and 13 patients were lost to follow-up within 6 months (Figure 2). Thus, the final study population consisted of 93 patients, of whom 13 patients with an elective PCI could finalize their DAPT at 6 months. Out of 80 patients continuing DAPT with a total treatment duration of 9–12 months, blood was drawn again at the second study visit (T2, 6 months) in 68 patients. Unfortunately, no TG could be measured in the other 12 patients due to logistical reasons. Baseline characteristics of patients lost to follow-up or with missing TG measurements at T2 did not differ from the final study population or the T2 population, respectively (Supplementary Tables 2, 3). During the treatment period, 14 patients (15.1%) suffered from clinically relevant bleeding (BARC score ≥2), of which 7 bleedings were documented at T1, 3 bleedings at T2, and 4 bleedings at T3 (Supplementary Table 1). No patient had more than one clinically relevant bleeding event during follow-up.

Baseline characteristics of the 93 included patients are listed in Table 1. Significant differences in prevalence of anemia and TIA/CVA in medical history were found between patients with and without clinically relevant bleeding. No significant differences were found in amount of days from PCI to T1 between both groups. Most of the patients (*n* = 80, 86.0%) used DAPT for 9–12 months after PCI of which 12 patients (15.0%) suffered from clinically relevant bleeding during treatment. Thirteen patients (14.0%) used DAPT for 6 months according to prescription after elective PCI, of whom three (20.0%) had a clinically relevant bleeding.

**Table 1:** Baseline characteristics.

	**All patients (*N* = 93), mean (±SD), number (%)**	**Patients with clinically relevant bleeding (*N* = 15), mean (±SD), number (%)**	**Patients without clinically relevant bleeding (*N* = 78), mean (±SD), number (%)**	**Significance (*p*-value)**
**Inclusion criteria**				
Female gender	49 (52.7%)	7 (46.7%)	42 (53.8%)	0.610
Age >75 years	49 (52.7%)	8 (53.3%)	41 (52.6%)	0.956
Age (years)	72.4 (9.0)	70.5 (8.6)	72.8 (9.1)	0.359
Weight <60 kg	6 (6.5%)	2 (13.3%)	4 (5.1%)	0.236
Hypertension	86 (92.5%)	12 (80.0%)	74 (86.9%)	0.080
Anemia	22 (23.7%)	8 (53.3%)	14 (17.9%)	0.003^*^
eGFR <60 ml/min	51 (54.8%)	9 (60.0%)	42 (53.8%)	0.661
Renal function (eGFR ml/min)	59.6 (20.1)	55.5 (20.7)	60.4 (20.0)	0.390
Known hepatitis or liver transplant	0 (0%)	0	0	–
TIA/CVA in medical history	24 (25.8%)	7 (46.7%)	17 (21.8%)	0.044^*^
Bleeding in medical history	8 (8.6%)	2 (13.3%)	6 (7.7%)	0.475
Diabetes mellitus	32 (34.4%)	4 (26.7%)	28 (35.9%)	0.491
NSAID usage	8 (8.6%)	1 (6.7%)	7 (9.0%)	0.770
SSRI usage	5 (5.4%)	0 (0.0%)	5 (6.4%)	0.313
Gastric ulcer/bleeding in medical history	15 (16.1%)	5 (33.3%)	10 (12.8%)	0.062
High-risk referral	14 (15.1%)	1 (6.7%)	13 (16.7%)	0.680
**Laboratory characteristics**				
Hemoglobin (g/dl)	13.2 (1.0)	12.9 (1.1)	13.4(1,0)	0.245
Hematocrit (L/L)	0.40 (0.04)	0.39 (0.05)	0,40 (0,04)	0.356
Thrombocytes (×10^9^/L)	252.0 (72.7)	252.9 (66.6)	251.8 (74.2)	0.958
Leukocytes (×10^9^/L)	7.5 (2.05)	6.9 (2.0)	7.6 (2.1)	0.217
PT (s)	10.5 (0.5)	10.7 (0.6)	10.5 (0.5)	0.113
aPTT (s)	26.3 (2.0)	26.2 (1.5)	26.3 (2.1)	0.821
**Patient characteristics**				
Smoking	15 (16.1%)	1 (6.7%)	14 (17.9%)	0.314
≥8 glasses of alcohol per week^**^	10 (10.8%)	2 (13.3%)	8 (10.3%)	0.346
Dyslipidemia	35 (37.6%)	7 (46.7%)	28 (35.9%)	0.430
Malignancy (active)	7 (7.5%)	3 (20.0%)	4 (5.1%)	0.080
Atrial fibrillation	4 (4.3%)	0 (0.0%)	4 (5.1%)	1.000
PPI usage	75 (80.6%)	14 (93.3%)	61 (78.2%)	0.174
ACS as PCI indication	59 (63.4%)	11 (73.3%)	48 (61.5%)	0.385
Interval PCI to T1 in days	54.7 (31.2)	65.9 (47.2)	52.5 (27.01)	0.128
**Antithrombotics**				
Aspirin	93 (100%)	15 (100%)	78 (100%)	–
Clopidogrel	69 (74.2%)	11 (73.3%)	58 (74.3%)	0.934
Ticagrelor	5 (5.4%)	1 (6.7%)	4 (5.1%)	0.809
Prasugrel	19 (20.4%)	3 (20.0%)	16 (20.5%)	0.964
**Treatment period**				
DAPT duration 6 months	13 (14.0%)	3 (20.0%)	10 (12.8%)	0.463
DAPT duration 9 to 12 months	80 (86.0%)	12 (80.0%)	68 (87.2%)	

*The meaning of the bold values with an asterisk is described in the subheading*

* *p < 0.05 is considered as significant*.

*N, number of patients; SD, standard deviations; BARC, bleeding academic research consortium; eGFR, estimated glomerular filtration rate; TIA, transient ischemic attack; CVA, cerebral vascular attack; NSAID, non-steroidal anti-inflammatory drug; SSRI, selective serotonin reuptake inhibitor; PT, prothrombin time; aPTT, activated partial thromboplastin time; PPI, proton pump inhibitor; ACS, acute coronary syndrome; PCI, percutaneous coronary intervention; DAPT, dual antiplatelet therapy*.

** *A cutoff value of ≥8 glasses of alcohol per week was chosen based on the HASBLED bleeding score (34)*.

### Thrombin Generation and Clinically Relevant Bleeding During Follow-Up

**Figure 3:**
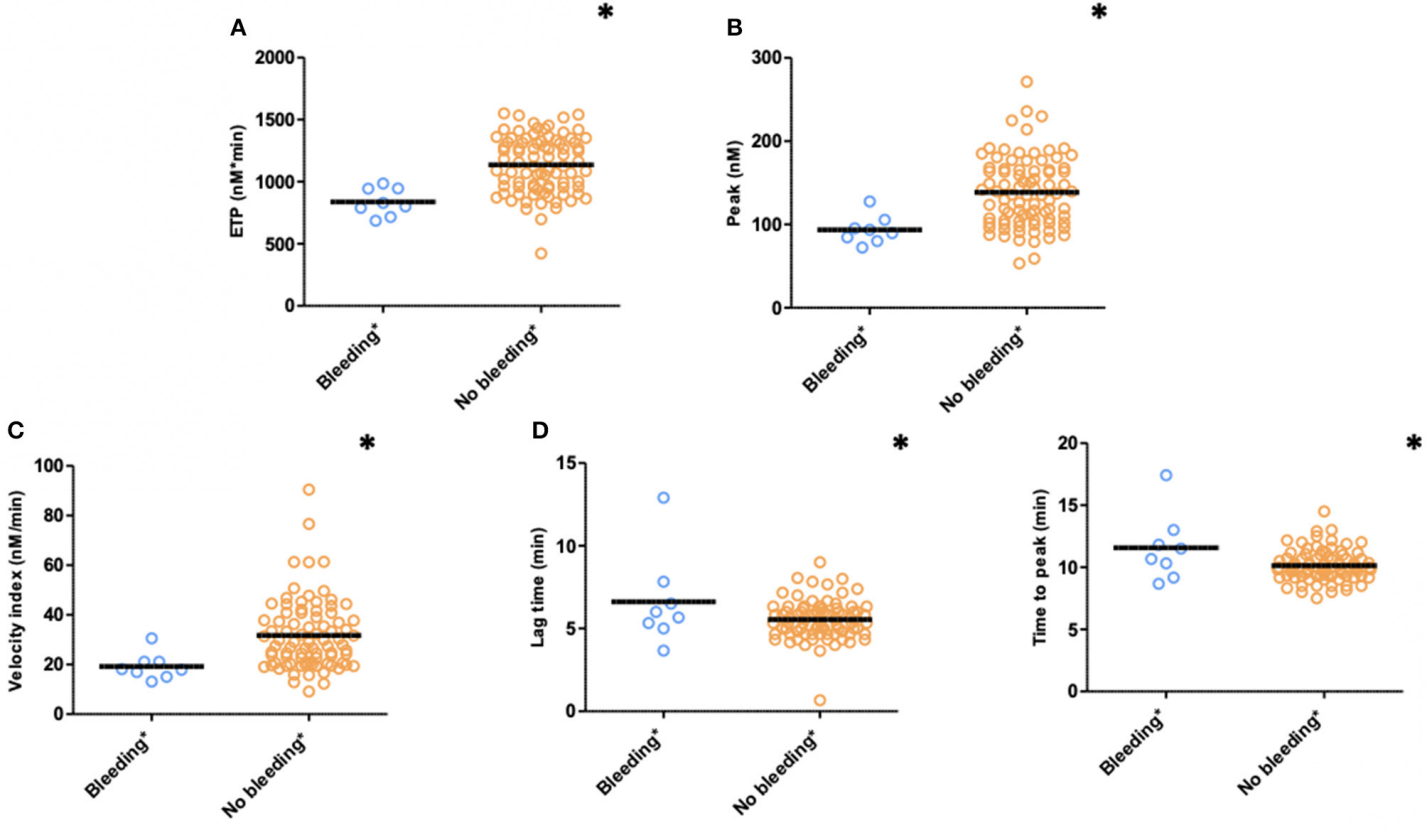
Thrombin generation (TG) in PPP patients with (n = 15) and without (n = 78) clinically relevant bleeding (N = 93) during follow-up (bleedings recorded at T2 and T3). **(A)** ETP, **(B)** Peak, **(C)** Velocity index, **(D)** Lag time, and **(E)** Time to peak. Significant lower ETP levels (p ≤ 0.001), peak height (p = 0.004), and velocity index (p = 0.016) in patients with clinically relevant bleeding during follow-up. Significant longer time to peak (p = 0.007) and lag time (p = 0.036) in patients with clinically relevant bleeding. Means are indicated by lines. *p < 0.05. ETP, Endogenous Thrombin Potential; PPP, Platelet Poor Plasma; N, number of patients.

TG measured 1 month after PCI (T1) was significantly lower with respect to ETP (*p* < 0.001), peak height (*p* = 0.004), and velocity index (*p* = 0.016) in plasma from patients with bleeding during follow-up (*n* = 8) compared to patients without bleeding complications (*n* = 85) (bleedings recorded at T2 and T3) (Figure 3). Moreover, patients with bleeding had a significantly longer time-to-peak (*p* = 0.007) and lag time (*p* = 0.036) compared to patients without bleeding (Figure 3). Similarly, at 6 months after PCI (T2, *n* = 68), significantly lower peak level (*p* = 0.039), velocity index (*p* = 0.031), and a trend toward a lower ETP (*p* = 0.072) were detected in plasma from patients who experienced bleeding during follow-up (*n* = 4) (bleedings recorded at T3) (Table 2).

**Table 2:** Thrombin generation 6 months after PCI in PPP for patients with and without clinically relevant bleeding during follow-up (recorded at T3).

	**Clinically relevant bleeding medians (IQR)**	**Non-clinically relevant bleeding medians (IQR)**	**p-value**
**Thrombin generation in PPP 1 pM (N = 68) (Bleeding N = 4, Non-bleeding N = 64)**			
ETP	807.18 (608.09–1,006.28)	1,030.15 (911.22–1149.08)	0.072
Peak	88.88 (63.79–113.97)	127.50 (104.24–150.76)	0.039^*^
Velocity index	18.61 (13.30–23.92)	27.24 (21.51–32.97)	0.031^*^
Lag time	5.34 (4.63–6.05)	5.33 (4.55–6.05)	0.734
Time to peak	10.17 (9.42–10.92)	10.17 (9.25–11.09)	0.575

* *A p < 0.05 was considered significant. PPP, platelet poor plasma; pM, picomolar; BARC, bleeding academic research consortium; IQR, interquartile range, N, number of patients, ETP, endogenous thrombin potential*.

### Coagulation Factors as Determinants of TG in PPP

At T1, ETP was significantly determined by FII activity level [Beta 8.675 (95% CI 1.336–16.014), *p* = 0.021], as expected, while the peak level was significantly determined by the FV activity level [Beta −0.663 (95% CI −1.244 to −0.083), *p* = 0.026]. ETP, peak level, and velocity index were all significantly affected by FVIII activity [Beta, respectively, 2.128 (95% CI 0.045–4.210), 0.516 (95% CI 0.156–0.877), and 0.171 (95% CI 0.052–0.291) (*p*-value, respectively, 0.045, 0.006, and 0.006)]. Lag time and time-to-peak were mainly determined by the plasma fibrinogen concentration [Beta, respectively, 0.688 (95% CI 0.119–1.258) and 0.788 (95% CI 0.270) (*p*-value, respectively, 0.019 and 0.004)] (Table 3).

**Table 3:** Coagulation factors as determinants of thrombin generation—multiple regression analysis.

	**ETP**	**Peak**	**Velocity index**	**Lag time**	**Time to peak**
**Beta (95% CI)**					
(Adjusted *R*^2^)	0.104	0.255	0.273	0.265	0.161
Fibrinogen	49.344 (−38.385–137.073)	5.050 (−10.132–20.233)	2.339 (−2.700–7.379)	0.788^*^ (0.270–1.370)	0.688^*^ (0.119–0.1258)
vWF activity	−0.174 (−1.930–1.582)	−0.017 (−0.320–0.287)	0.006 (−0.095–0.107)	0.005 (−0.005–0.015)	0.004 (−0.008–0.015)
Factor II activity	8.675^*^ (1.336–16.014)	0.666 (−0.604–1.936)	0.099 (−0.323–0.520)	−0.025 (−0.068–0.019)	−0.007 (−0.055–0.040)
Factor V activity	−2.220 (−5.574–1.134)	−0.663^*^ (−1.244– −0.083)	−0.181 (−0.374–0.011)	0.018 (−0.002–0.038)	0.021 (0.000–0.043)
Factor VII activity	−0.977 (−4.454–2.500)	0.031 (−0.571–0.632)	0.030 (−0.169–0.230)	−0.008 (−0.029–0.120)	−0.011 (−0.033–0.012)
Factor VIII activity	2.128^*^ (0.045–4.210)	0.516^*^ (0.156–0.877)	0.171^*^ (0.052–0.291)	−0.003 (−0.015–0.009)	−0.008 (−0.022–0.005)
Factor IX activity	−1.120 (−5.125–2.885)	0.012 (−0.681–0.705)	0.013 (−0.218–0.243)	−0.003 (−0.027–0.020)	−0.002 (−0.028–0.024)
Factor X activity	−1.629 (−6.824–3.567)	0.051 (−0.849–0.950)	0.115 (−0.184–0.413)	0.026 (−0.005–0.057)	0.011 (−0.023–0.045)
Factor XI activity	−1.665 (−3.876–0.546)	−0.219 (−0.601–0.164)	−0.059 (−0.186–0.068)	0.009 (−0.004–0.022)	0.008 (−0.006–0.022)
Factor XII activity	1.149 (−1.631–3.928)	0.083 (−0.398–0.564)	−0.030 (−0.190–0.129)	−0.007 (−0.024–0.009)	−0.005 (−0.023–0.013)

* *Indicates a p < 0.05, considered as significant*.

*CI, confidence interval; ETP, endogenous thrombin potential; vWF, von willebrand factor*.

## Discussion

The main aim of our study was to investigate whether TG might have potential to identify patients with an increased bleeding risk during the following DAPT treatment period of 6–12 months, by comparing the TG parameters between “bleeders” and “non-bleeders.” TG parameters measured in PPP, at 1 and 6 months after PCI, showed (significant) lower ETP, peak, and velocity index levels in patients with a clinically relevant bleeding episode compared to patients without bleeding during follow-up.

Since thrombin generation was performed in the absence of platelets, the observed differences between patients with and without bleeding might be explained by variation in coagulation factors. Following this theory, impaired thrombin generation resulting from one or several coagulation factor levels in the low-normal range, on top of DAPT, could ultimately contribute to an increased risk of bleeding. Mean values of all coagulation factors measured in this study were within normal range. We showed in this study that ETP was mostly determined by prothrombin levels, and peak height by FV level, as confirmed by previous studies (24, 35–37). ETP, peak height, and velocity index were all significantly determined by the level of factor VIII. This positive relation can be explained by the strong feedback loop between thrombin and factor VIII (37, 38). Higher ETP in case of higher factor II (prothrombin) levels was expected, since factor II is the precursor of thrombin and has been confirmed in previous research (24, 36, 39). We found a negative association between FV activity and peak height. Interestingly, this inverse relationship was also found in healthy individuals in a previous publication by Dielis et al. (36). This negative relation might be attributed to the fact that factor V has both procoagulant and anticoagulant potential; activated FV functions in the procoagulant pathways, but in its inactivated form, FV acts as a cofactor for activated protein C (APC) in the regulation of FVIIIa (40). Increased lag time and time-to-peak were both associated with higher levels of fibrinogen. Based on other studies investigating TG in normal and defibrinated plasma, this can be explained by the ability of fibrinogen/fibrin to bind thrombin and subsequently inhibit thrombin-mediated FVIII activation (24, 36, 41, 42). In the presence of DAPT, such effect may obviously increase the risk of bleeding too. Besides coagulation factors, circulating cell-derived microparticles also play a role in thrombogenesis (43). However, in this study we did not focus on the contribution of these microparticles to TG potential. In this study, we have shown that TG potential is dependent on multiple coagulation factors and that lower activity of various coagulation factors can lead to impaired TG. This indicates that TG could help in the identification of patients with a high bleeding risk, along with the assessment of other risk factors that are associated with bleeding such as presented in Table 1.

A key strength of the present study is the use of state-of-the-art TG in a real-life setting with prospective documentation of bleeding complications, according to internationally accepted criteria (BARC 2 and 3 bleedings), an important patient-centric outcome with impact on the patients' quality of life. Moreover, we studied high-risk patients receiving DAPT, representing a challenging and complex patient group in whom both bleeding complications and recurrent ischemic events frequently occur. However, several limitations to this study also need to be acknowledged. First, the study population was small, especially in certain subgroups. Nevertheless, the sample size was large enough to find clinically relevant and significant differences between groups. Another limitation is the lost to follow-up of 13 patients within 6 months and missing TG data of 12 patients at T2. However, selection bias is considered unlikely since no relevant differences in baseline characteristics were found between these patients and the included study population (Supplementary Tables 2, 3) and no patients were lost to follow-up because of bleeding or death (as checked with their general practitioner and the hospital's electronic medical records). Next, baseline characteristics of the study population showed (significant) differences in prevalence of anemia, TIA/CVA, and malignancy between patients with and without clinically relevant bleeding. Unfortunately, subgroups were too small to perform a multivariate analysis to adjust for these possible confounders. Although anemia at the time of PCI was found more frequently in the bleeding group, hemoglobin levels at the time of TG measurements were comparable between groups (12.9 and 13.4 g/dl for bleeders and non-bleeders, respectively, *p* = 0.245). Therefore, it seems unlikely that the hemoglobin levels have affected the differences in TG parameters between groups. TIA/CVA and malignancy are, among others, well-known risk factors for increased bleeding risk after PCI, as was also stated in the recent Academic Research Consortium for High Bleeding Risk (ARC-HBR) consensus document (44). Thus, differences between bleeders and non-bleeders in incidence of these factors were to be expected. Previous studies have shown both negative and positive associations between TIA/CVA and TG parameters, making it difficult to speculate about the possible impact on TG parameters of baseline differences in previous stroke (45, 46). Lastly, it would have been interesting to investigate the effect of DAPT on TG in PRP or whole blood as well. Nevertheless, we have deliberately measured TG in PPP, since this is by far the most used and standardized method of TG measurement and offers the opportunity to rule out the possible role of platelets in this setting.

## Conclusion

High clinical-risk patients with DAPT with clinically relevant bleeding during follow-up have (significantly) lower ETP levels, peak height, and velocity index in PPP compared to patients without bleeding, both at 1 and at 6 months after PCI. We have shown that this might at least be partly explained by variation in coagulation factors. Relatively low thrombin generation in these patients acts as a “second hit,” on top of DAPT, thus increasing the bleeding risk. TG performed in PPP may have the potential to aid in the identification of patients with an increased bleeding risk during DAPT. However, more research, with a larger sample size, would be needed to determine cutoff values for TG to be able to adequately identify these patient groups. Furthermore, future research could investigate the capability of other TG-based assays to identify patients with increased bleeding risk during DAPT, e.g., the potential benefit of PRP and/or additional measurement of microparticles.

## Data Availability Statement

The raw data supporting the conclusions of this article will be made available by the authors, without undue reservation.

## Ethics Statement

The studies involving human participants were reviewed and approved by METC aZM/UM approval number: 11-2-096. The patients/participants provided their written informed consent to participate in this study.

## Author Contributions

CB and SZ designed and conceptualized the study. CB wrote the concept of the article, on direct supervision of SZ, HC, and RHO. MV and PM commented on the draft version of the article. CB coordinated all aspects of this research. RGO and YH directed the multiple laboratory assessments. MV, SZ, RHO, HC, CB, LV, and AH were involved in inclusion of patients. All authors contributed to the article and approved the submitted version.

## Conflict of Interest

The authors declare that the research was conducted in the absence of any commercial or financial relationships that could be construed as a potential conflict of interest.
