# Supplementary material for: Thrombin Generation as a Method to Identify the Risk of Bleeding in High Clinical-Risk Patients Using Dual Antiplatelet Therapy
Source: Front Cardiovasc Med. 2021 Jun 10;8:679934. doi: 10.3389/fcvm.2021.679934 (PMC8224526; doi:10.3389/fcvm.2021.679934)
Supplement: Supplementary file 1 [file Data_Sheet_1.docx]

| *Supplementary table 1. Most severe bleeding event during treatment period at T1, T2 and T3* | | | |
| --- | --- | --- | --- |
|  | **T1, number (%)** | **T2, number (%)** | **T3, number (%)** |
| No bleeding | 74 (79.6%) | 71 (76.3%) | 53 (77.9%) |
| BARC 1 | 12 (12.9%) | 19 (20.4%) | 11 (16.2%) |
| BARC 2 | 6 (6.5%) | 3 (3.2%) | 4 (5.9%) |
| BARC 3 | 1 (1.1%) | 0 (0%) | 0 (0%) |
| Total (N) | 93 | 93 | 68 |
| *T1 = consultation 1 month after PCI, T2 = consultation 6 months after PCI, T3 = consultation 12 months after PCI, PCI = Percutaneous Coronary Intervention, N = number of patients, BARC = Bleeding Academic Research Consortium* | | | |

| *Supplementary table 2.  Baseline characteristics from patients included in this study (N=93) compared with patients lost to follow-up within 6 months (N=13)* | | | |
| --- | --- | --- | --- |
|  | **Patients included in study** (N=93) Mean (±SD), number (%) | **Patients lost to follow-up <6months** (N=13) Mean (±SD), number (%) | **Significance** (p-value) |
| *Inclusion criteria* | | | |
| Female gender | 49 (52.7%) | 6 (46.2%) | 0.660 |
| Age >75 years | 49 (52.7%) | 9 (69.2%) | 0.264 |
| Age (years) | 72,4 (9.0) | 76.4 (11.5) | 0.155 |
| Weight <60 kg | 6 (6.5%) | 1 (7.7%) | 0.867 |
| Hypertension | 86 (92.5%) | 9 (69.2%) | **0.010*** |
| Anemia | 22 (23.7%) | 2 (15.4%) | 0.506 |
| eGFR <60 ml/min | 51 (54.8%) | 3 (23.1%) | **0.033*** |
| Renal function (eGFR ml/min) | 59.6 (20.1) | 68.3 (18.7) | 0.145 |
| Known hepatitis or liver transplant | 0 (0.0%) | 0 (0.0%) | 1.000 |
| TIA/CVA in medical history | 24 (25.8%) | 4 (30.8%) | 0.705 |
| Bleeding in medical history | 8 (8.6%) | 1 (7.7%) | 0.913 |
| Diabetes mellitus | 32 (34.4%) | 7 (53.8%) | 0.175 |
| NSAID usage | 8 (8.6%) | 1 (7.7%) | 0.913 |
| SSRI usage | 5 (5.4%) | 2 (15.4%) | 0.176 |
| Gastric ulcer /bleeding in medical history | 15 (16.1%) | 0 (0%) | 0.120 |
| High risk referral | 14 (15.1%) | 0 (0%) | 0.136 |
|  | | | |
| *Laboratory characteristics* | | | |
| Hemoglobin(g/dL) | 13.2 (1.61) | 13.99(1.68) | 0.128 |
| Hematocrit (L/L) | 0.40 (0.04) | 0,42 (0.05) | 0.090 |
| Thrombocytes (x10^9^/L) | 252.0 (72.7) | 251.1 (41.3) | 0.967 |
| Leukocytes (x10^9^/L) | 7.5 (2.05) | 7.8 (1.9) | 0.631 |
| PT (sec) | 10.5 (0.5) | 10.6 (0.5) | 0.826 |
| aPTT (sec) | 26.3 (2.0) | 26.1 (2.6) | 0.709 |
|  | | | |
| *Patient characteristics* | | | |
| Smoking | 15 (16.1%) | 4 (30.8%) | 0.207 |
| ≥8 glasses alcohol per week** | 10 (10.8%) | 3 (23.1%) | 0.133 |
| Dyslipidemia | 35 (37.6%) | 3 (23.1%) | 0.308 |
| Malignancy (active) | 7 (7.5%) | 1 (7.7%) | 0.983 |
| Atrial fibrillation | 4 (4.3%) | 0 (0.0%) | 0.448 |
| PPI usage | 75 (80.6%) | 10 (76.9%) | 0.837 |
| ACS as PCI indication | 59 (63.4%) | 8 (61.5%) | 0.895 |
| Interval PCI to T1 in days | 54.7 (31.2) | 59.5 (39.2) | 0.149 |
|  | | | |
| *Antithrombotics* | | | |
| Aspirin | 93 (100%) | 12 (100%) | - |
| Clopidogrel | 69 (74.2%) | 12 (92.3%) | 0.152 |
| Ticagrelor | 5 (5.4%) | 1 (7.7%) | 0.736 |
| Prasugrel | 19 (20.4%) | 0 (0.0%) | 0.073 |
|  | | | |
| ** a p value <0.05 was considered significant*  *N=number of patients, SD = Standard Deviations, BARC = Bleeding Academic Research Consortium, eGFR = estimated Glomerular Filtration Rate,  TIA = Transient Ischemic Attack, CVA = cerebral vascular attack, NSAID = Nonsteroidal Anti-Inflammatory Drug, SSRI = Selective Serotonin Reuptake Inhibitor, PT = Prothrombin Time, aPTT = Activated Partial Thromboplastin Time, PPI=Proton Pump Inhibitor, ACS = Acute Coronary Syndrome, PCI = Percutaneous Coronary Intervention, DAPT = Dual Antiplatelet Therapy ** A cut-off value of ≥8 glasses alcohol per week was chosen based upon the HASBLED bleeding score. [46]* | | | |

| *Supplementary table 3.  Baseline characteristics from patients with TG measured at T1 and T2 compared with patients with missing TG data at T2, both using DAPT >6 months* | | | |
| --- | --- | --- | --- |
|  | **Patients with TG at T1 and T2** (N=68) Mean (±SD), number (%) | **Patients with missing TG at T2** (N=12) Mean (±SD), number (%) | **Significance** (p-value) |
| *Inclusion criteria* | | | |
| Female gender | 38 (55.9%) | 7 (58.3%) | 0.875 |
| Age >75 years | 31 (45.6%) | 7 (58.3%) | 0.418 |
| Age (years) | 71.7 (8.1) | 71.4 (13.1) | 0.910 |
| Weight <60 kg | 6 (8.8%) | 0 (0.0%) | 0.288 |
| Hypertension | 64 (94.1%) | 12 (100%) | 0.392 |
| Anemia | 14 (20.6%) | 2 (16.7%) | 0.756 |
| eGFR <60 ml/min | 38 (55.9%) | 6 (50.0%) | 0.707 |
| Renal function (eGFR ml/min) | 61.1 (19.5) | 52.8 (24.6) | 0.192 |
| Known hepatitis or liver transplant | 0 (0.0%) | 0 (0.0%) | 1.000 |
| TIA/CVA in medical history | 15 (22.1%) | 4 (33.3%) | 0.400 |
| Bleeding in medical history | 6 (8.8%) | 2 (16.7%) | 0.407 |
| Diabetes mellitus | 25 (36.8%) | 3 (33.3%) | 0.821 |
| NSAID usage | 6 (8.8%) | 1 (8.3%) | 0.956 |
| SSRI usage | 4 (5.9%) | 0 (0.0%) | 0.392 |
| Gastric ulcer /bleeding in medical history | 13 (19.1%) | 1 (8.3%) | 0.368 |
| High risk referral | 10 (14.7%) | 3 (25%) | 0.376 |
|  | | | |
| *Laboratory characteristics* | | | |
| Hemoglobin(g/dL) | 13.36 (1.69) | 12.9 (1.42) | 0.486 |
| Hematocrit (L/L) | 0.40 (0.05) | 0,39 (0.04) | 0.550 |
| Thrombocytes (x10^9^/L) | 244.2 (68.2) | 293.8 (103.4) | 0.134 |
| Leukocytes (x10^9^/L) | 7.2 (1.8) | 8.2 (2.1) | 0.121 |
| PT (sec) | 10.5 (0.5) | 10.7 (0.5) | 0.186 |
| aPTT (sec) | 26.2 (2.1) | 26.5 (1.7) | 0.716 |
|  | | | |
| *Patient characteristics* | | | |
| Smoking | 13 (19.1%) | 1 (8.3%) | 0.115 |
| ≥8 glasses alcohol per week** | 6 (8.8%) | 1 (8.3%) | 0.988 |
| Dyslipidemia | 28 (41.2%) | 3 (25.0%) | 0.292 |
| Malignancy (active) | 5 (7.4%) | 1 (8.3%) | 0.906 |
| Atrial fibrillation | 4 (5.9%) | 0 (0.0%) | 0.392 |
| PPI usage | 53 (77.9%) | 11 (91.7%) | 0.276 |
| ACS as PCI indication | 45 (66.2%) | 5 (41.7%) | 0.108 |
| Interval PCI to T1 in days | 51.5 (24.6) | 52.3 (26.9) | 0.915 |
|  | | | |
| *Antithrombotics* | | | |
| Aspirin | 68 (100%) | 12 (100%) | - |
| Clopidogrel | 50 (73.5%) | 7 (58.3%) | 0.287 |
| Ticagrelor | 4 (5.9%) | 1 (8.3%) | 0.748 |
| Prasugrel | 14 (20.6%) | 4 (33.3%) | 0.333 |
|  | | | |
| ** a p value <0.05 was considered significant*  *N=number of patients, SD = Standard Deviations, BARC = Bleeding Academic Research Consortium, eGFR = estimated Glomerular Filtration Rate,  TIA = Transient Ischemic Attack, CVA = cerebral vascular attack, NSAID = Nonsteroidal Anti-Inflammatory Drug, SSRI = Selective Serotonin Reuptake Inhibitor, PT = Prothrombin Time, aPTT = Activated Partial Thromboplastin Time, PPI=Proton Pump Inhibitor, ACS = Acute Coronary Syndrome, PCI = Percutaneous Coronary Intervention, DAPT = Dual Antiplatelet Therapy ** A cut-off value of ≥8 glasses alcohol per week was chosen based upon the HASBLED bleeding score. [44]* | | | |
